# At what age should the Uyghur minority initiate cervical cancer screening if screened using careHPV

**DOI:** 10.1002/cam4.4409

**Published:** 2021-11-24

**Authors:** Guzhalinuer Abulizi, Patiman Mijiti, Gulimire Naizhaer, Gulixian Tuerxun, Guzhanuer Abuduxikuer, Yuan‐Yuan Zhang, Hua Li, Tangnuer Abulimiti, Guligeina Abudurexiti, Kailibinuer Aierken, Ling Lu, Anaerguli Maimaiti

**Affiliations:** ^1^ 5th Department of Gynecology Affiliated Tumor Hospital of Xinjiang Medical University Urumqi China; ^2^ 3rd Department of Gynecology Affiliated Tumor Hospital of Xinjiang Medical University Urumqi China

**Keywords:** careHPV, cervical cancer, CIN2+, screening

## Abstract

**Background:**

The careHPV test as a primary screening method for cervical cancer has been proven to be the best option for Uyghur women in Xinjiang in a previous study. In this research, we aim to discuss the appropriate age for Uyghur women in Xinjiang to be screened for cervical cancer using careHPV.

**Methods:**

Eleven thousand women aged 20–69 years old (mean age 38.93 ± 9.74) from South Xinjiang were screened using careHPV and liquid‐based cytology, and the positive results were referred for colposcopy and cervical biopsy. A questionnaire regarding basic social characteristics, sexual practices, and reproductive history was administered to each woman. The age‐specific prevalence of HPV positivity, cytology abnormality, and cervical intraepithelial neoplasia (CIN) 2+ in ≥25, ≥30, and ≥35 age groups were analyzed, and the diagnostic value of careHPV in the three age groups was evaluated. The chi‐squared test was used to compare the differences between age groups. The sensitivity, specificity, positive predictive value, negative predictive value, and area under the receiver operating characteristic curve were calculated.

**Results:**

The women were mostly married (76.3%) and delivered at 15–19 years of age (61.4%). The HPV infection rate was 9.15% and detection rates of CIN2+ and invasive cervical cancer were 1.53% (1530/100,000) and 0.25% (250/100,000), respectively. The first peak of HPV(+) appeared at the age of 30–34, while CIN2+ appeared at 35–39. CareHPV performed similarly well in the three age groups.

**Conclusion:**

Based on the results of our study, Uyghur women in Xinjiang should be recommended to initiate cervical cancer screening at the age of 30 years when screened using careHPV.

## Graphical abstract

Cervical cancer screening should be started at 30 years for Uyghur minority when adopting the careHPV as primary screening method.

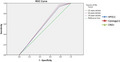

## 1 INTRODUCTION

In 2018, 570,000 women were diagnosed with cervical cancer worldwide, resulting in 311,000 deaths. Furthermore, nearly 90% of these new cases occurred in developing countries.^1^ Screening remains to be the predominant preventive method for cervical cancer even after the roll out of the HPV vaccine worldwide. Since 2009, free cervical cancer screening has been available for rural women aged 35–59 years under a government‐sponsored program proposed by the Ministry of Health. Visual inspection with acetic acid (VIA) or Pap smears has been adopted as the primary screening method in two cancer screening programs. Around 100,000 women from rural areas benefit from the free screening policy each year.^2^ However, according to the data from the Sixth National Population Census, the total number of women aged 35–64 in rural areas is about 1.6 billion.^3^

Pap smear and visual inspection with acetic acid/Lugol's iodine (VIA/VILI) showed poor effectiveness in practical cervical cancer screening projects. To add to that, the screening of hundreds of millions of Chinese women remains the biggest challenge for China. More studies are required to offer more reliable evidence for policymakers to select the optimum strategy based on the corresponding health resources.^4^

Cervical cancer is the most common cancer in Uyghur women, a minority group living in Western China. A survey that investigated cervical cancer mortality in eight ethnic minorities in China showed the highest mortality in the Uyghur (17.3/105), and the mortality rate was much higher in Uyghur women in every age group than in the Miao and Yi women.^5^

Cervical cancer screening policy is different all around the world given the inequality of health resources, and different recommendations exist on the initiating age for screening, ranging from 25 years old to 35 years old when using the human papillomavirus (HPV) method. The American Society for Colposcopy and Cervical Pathology guidelines for cervical cancer screening suggest the use of a co‐testing strategy, an HPV test along with cytology, for women older than 30 years.^6^ The World Health Organization recommends that women between the ages of 35 and 40 years in low‐resource settings should be screened at least once with cervical cytology, HPV DNA testing, or VIA.^7^

It is still controversial which type of screening protocol should be implemented in a low‐resource region and which age should be the starting age of screening among women with a substantial rate of early marriage. Our research team concluded from a large population‐based screening study that primary screening through careHPV with liquid‐based cytology (LBC) triage test is the best option for the Xinjiang region,^8^ and careHPV showed acceptable consistency with the Hybrid Capture 2 test in detecting HPV infection.^9^ Based on the promising performance of careHPV, we attempted to discuss the reasonable age to initiate the cervical cancer screening in Uyghur women and provide relevant evidence for cervical cancer screening guidelines in China.

## 2 MATERIALS AND METHODS

### 2.1 Study populations

This was a population‐based, cross‐sectional study with a total of 11,000 women being recruited from Kashgar Maralbexi County and Hotan Karakax County, Xinjiang Uyghur Autonomous Region, China, between May 2013 and May 2014. Eligible women were aged 20–69 years (mean age 38.93 ± 9.74; divided into ten 5‐year age groups, wherein a total of 77.8% of the women were aged 25–44), sexually active, not pregnant, and had no history of cervical intraepithelial neoplasia (CIN), cervical cancer, or hysterectomy. Demographic and lifestyle data including age, age at first sexual intercourse, education, income, menstrual status, parity, and number of deliveries were obtained using a questionnaire. All participants signed a written consent form in their native language prior to participation in the study. The study protocol was approved by the Ethics Committee of The Affiliated Tumor Hospital of Xinjiang Medical University.

### 2.2 Screening tests

Eleven thousand women were recruited at Karakax Maternal and Child Care Hospital, Hotan, China and Maralbexi Maternal and Child Care Center, Kashgar, China. First, a cervical brush sample was obtained to process the LBC (Triplex International Bioscience [China] Co. Ltd.) sample and the results were expressed according to the Bethesda classification. A second cervical specimen was taken with a brush to perform the careHPV^TM^ test (Qiagen Inc.), which was used for the identification of HPV cervical infections. The careHPV test is an in vitro nucleic acid hybridization assay coupled with signal amplification that uses microplate chemiluminescence for the qualitative detection of 14 types of HR‐HPV (16, 18, 31, 33, 35, 39, 45, 51, 52, 56, 58, 59, 66, and 68) in cervical tissue specimens. The careHPV test was performed onsite within 1 day by well‐trained postgraduate medical students in the County Health Center for Women and Children. Women with positive results on LBC or careHPV were re‐called for a colposcopy examination 1 week after the primary screening. A standardized colposcopy examination was performed by gynecologic oncologists at the Affiliated Tumor Hospital of the Xinjiang Medical University. Histologic findings were classified as follows: no dysplasia, low‐grade dysplasia (CIN 1), high‐grade dysplasia (CIN 2–3), and squamous cancer cell (SCC).

### 2.3 Statistical analysis

Demographic information such as age, marital status, educational level, occupation, and sexual‐related status was quantified. The accuracy of four different screening strategies was evaluated by indicators of sensitivity, specificity, positive and negative predictive values, and receiver operating characteristic (ROC) curves. The chi‐squared test was used to compare the screening performance between strategies. SPSS software (version 10.0) was used to analyze the data. Statistical significance was assessed using two‐tailed tests with an α level of 0.05.

## 3 RESULTS

### 3.1 Participant's characteristics

Nearly half of the participants married only once, and 64.5% had delivered more than two children. Women preferred intrauterine devices (60.6%) for contraception compared to condoms (7.9%) and oral contraceptives (6.8%) (Table 1).

**TABLE 1:** Demographic characteristics of Uyghur women screened for cervical cancer (*n* = 11,000)

Characteristic	*N*	%
Age		
20–24	826	7.5
25–29	1289	11.7
30–34	1753	15.9
35–39	1809	16.4
40–44	2281	20.7
45–49	1428	13.0
50–54	883	8.0
55–59	508	4.7
60–64	161	1.5
≥65	62	0.6
Educational level		
Illiterate	1516	13.8
Primary	6155	55.9
Secondary	2836	25.8
Higher	493	4.5
Occupation		
Farmer	9733	88.5
Non‐farmer	1267	11.5
Marriage times		
1	5563	50.6
2–3	4797	43.6
>3	640	5.8
Parity		
≤2	3904	35.5
>2	7096	64.5
Contraception		
Condom	870	7.9
Intrauterine device	6662	60.6
Sterilization	988	9.0
Oral contraceptive	747	6.8
Other	1733	15.7

### 3.2 Age of first marriage and first delivery

**FIGURE 1:**
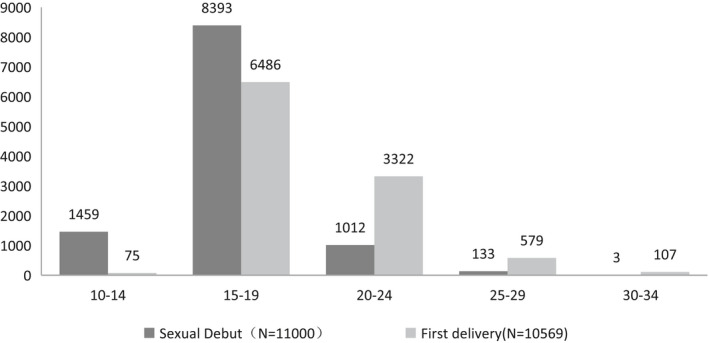
Age of Uyghur women at sexual debut and first delivery

All the women were married and delivered when they were older than 10 years old but younger than 34 years old. The age groups were divided by 5‐year intervals. The results show that Uyghur women in Xinjiang mostly got married and delivered their babies at 15–19 years of age (76.3% and 61.4%, respectively), with the second peak age for marriage at 10–14 years of age (Figure 1).

### 3.3 Age prevalence of HPV, cytology, and CIN2+ detection rate

Among 11,000 women, 1006 were infected with HPV, garnering an infection rate of 9.15% (Table 2). A total of 429 (3.9%) women were tested for abnormal cytology, including 33 women with atypical glandular cells of uncertain significance (AGC), 144 with atypical squamous cell of undetermined significance (ASC‐US), 64 with atypical squamous cells‐cannot exclude high‐grade squamous intraepithelial lesion (ASC‐H), 177 with low grade squamous intraepithelial lesion (LSIL), 67 with high grade squamous intraepithelial lesion (HSIL), and 4 with squamous cell carcinoma (SCC). Women with positive results for any of the two tests were referred for colposcopy. Cervical biopsy was performed on 1380 women, and according to the final histology results, the number of women who were classified as normal, CIN1, CIN2, CIN3, SCC, and adenocarcinoma were 1064, 147, 61, 79, 26, and 2, respectively. One woman was diagnosed with cervical tuberculosis and was excluded from the study. The detection rates of CIN2+ and invasive cervical cancer were 1.53% (1530/100,000) and 0.25% (250/100,000), respectively.

**TABLE 2:** Detection rate of HPV, cytology, and CIN2+ of Uyghur women when stratified by different age groups

Age group	HPV(+)	Cyto(+)^a^	CIN2+	HPV(+)/Cyto(−)^a^	HPV(+)/Cyto(+)^a^
<25 (*N* = 826)	66 (7.99%)	24 (2.91%)	5 (0.61%)	54 (6.54%)	12 (1.45%)
≥25 (*N* = 10174)	940 (9.24%)	405 (3.98%)	162 (1.59%)	692 (6.80%)	248 (2.44%)
	χ^2^ = 1.434 *p* = 0.231	χ^2^ = 2.356 *p* = 0.125	χ^2^ = 5.736 *p* = 0.017	χ^2^ = 2.164 *p* = 0.141
<30 (*N* = 2115)	167 (7.90%)	59 (2.79%)	17 (0.80%)	132 (6.24%)	35 (1.65%)
≥30 (*N* = 8885)	839 (9.44%)	370 (4.16%)	150 (1.69%)	614 (6.91%)	225 (2.53%)
	χ^2^ = ^2^4.920 *p* = 0.027	χ^2^ = 8.614 *p* = 0.003	χ^2^ = 9.684 *p* = 0.002	χ^2^ = 2.495 *p* = 0.114
<35 (*N* = 3868)	319 (8.25%)	107 (2.77%)	33 (0.85%)	261 (6.75%)	58 (1.50%)
≥35 (*N* = 7132)	687 (9.63%)	322 (4.51%)	134 (1.88%)	485 (6.80%)	202 (2.83%)
	χ^2^ = 5.794 *p* = 0.016	χ^2^ = 20.459 *p* = 0.000006092	χ^2^ = 14.549 *p* = 0.0001365	χ^2^ = 14.313 *p* = 0.000155
Total	1006 (9.1%)	429 (3.9%)	167 (1.5%)	746 (6.8%)	260 (2.4%)

Abbreviation: ASC‐US, atypical squamous cell of undetermined significance; CIN, cervical intraepithelial neoplasia; HPV, human papillomavirus.

^a^ ASC‐US was used as the cut‐off for cytology positive results.

**FIGURE 2:**
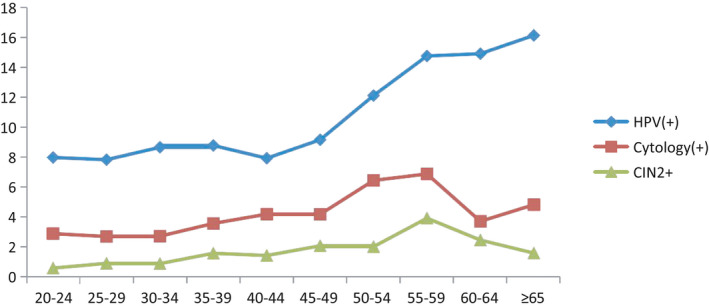
Age‐specific prevalence of HPV, cytology, and CIN2+ detection rate. CIN, cervical intraepithelial neoplasia; HPV, human papillomavirus.

When stratified by age, HPV detection rate kept increasing from 25 until 39 years of age, then showed a slight decline at age 40–44. It then sharply increased from 45 years old with no drop until the age ≥65 years. In contrast to HPV, cytological abnormalities showed an increasing trend later (starting at 30 years of age) and reached a peak at 55–59 years. The detection rate of CIN2+ also showed an increasing trend with increasing age starting from 20 years old, then a sharp increase appearing after the 30–34 age group, and reaching a peak at age 55–59, which is consistent with the cytology peak, and started to decline after that (χ^2^: 9.198, *p*: 0.002) (Figure 2).

### 3.4 careHPV performance by different age groups

**FIGURE 3:**
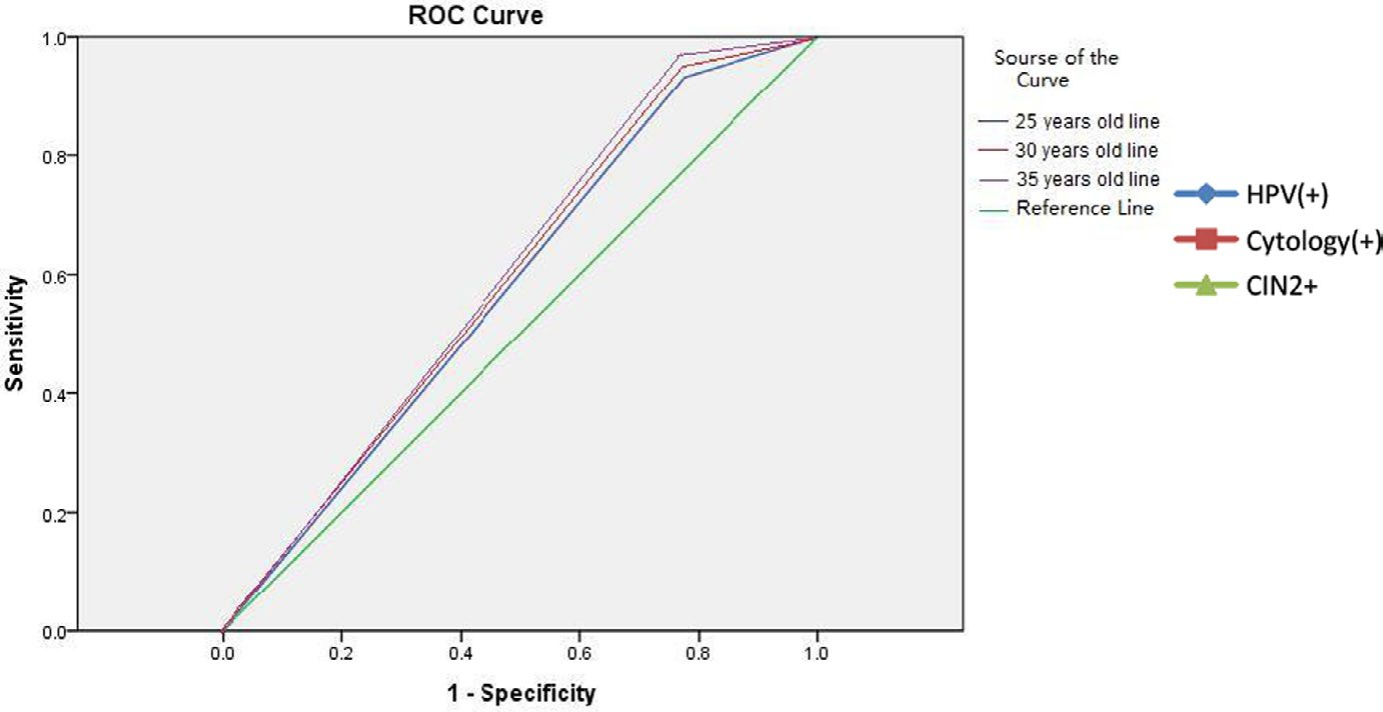
ROC curve of careHPV test by different starting age for screening. ROC, receiver operating characteristic

We evaluated the diagnostic value of the careHPV test using different starting ages (25, 30, 35). Sensitivity, specificity, positive predictive value (PPV), and negative predictive value (NPV) were similar in all groups, and the best performance was observed in women older than 35 years, with the highest sensitivity (93.1%), specificity (23.8%), PPV (29.4%), and NPV (94.8%). The area under the ROC curve was 0.593 for women older than 35 years, compared to 0.580 for women older than 30 years and 0.578 for women older than 25 years (Table 3, Figure 3).

**TABLE 3:** Performance of careHPV on different age groups among Uyghur women

Age group	Sensitivity%	Specificity%	PPV%	NPV%	AUC	SE	*p*	95% CI of AUC	
≥25	92.0	22.4	25.6	93.2	0.578	0.024	0.002	0.532	0.625
≥30	91.7	22.7	27.0	93.3	0.580	0.025	0.003	0.531	0.629
≥35	93.1	23.8	29.4	94.8	0.593	0.026	0.001	0.541	0.644

Abbreviations: AUC, area under the curve; CI, confidence interval; HPV, human papillomavirus; NPV, negative predictive value; PPV, positive predictive value; SE, standard error.

## 4 DISCUSSION

In China, wide disparities in cervical cancer incidence and mortality exist, and are mainly attributable to the large population and unequal economic development in different areas across the country. The majority of Chinese women have never been screened, and this is particularly true for women in rural areas with poor access to health resources. The Xinjiang Uyghur Autonomous Region, which is located in Western China, is a multiethnic area with a less developed economy. Uyghur women have the highest mortality and incidence of cervical cancer among 13 minorities who lived in Xinjiang. This is due to some high‐risk lifestyles related to HPV infection, which generally exist in Uyghur women, such as getting married and giving birth at an early age, multiple instances of pregnancy and delivery, poor genital hygiene, etc., which are believed to be closely related to cervical cancer.^10^ The mean age of the women (38.93 ± 9.74 years old) who participated in the study showed that they belonged to a high‐risk group for cervical cancer. The majority (81.7%) of the participants were elementary school and secondary school graduates, which is lower than the data of Na's^11^ (75.2%), and 88.5% of all women were farmers. Low educational level is an important risk factor for cervical cancer because it prevents women from accessing health information and existing health services due to lower awareness. Among the screened women, the rate of condom use is very low (7.9%), which is slightly higher than the overall rate (6.6%) of condom use among Chinese women from rural China but lower than that in Turkey (20.9%).^11^, ^12^

Studies on HPV prevalence and incidence indicate that the most consistent predictor of infection is sexual activity, particularly the age at first sexual intercourse or “sexual debut.” Of the women in this study, 76.3% were married by 15–19 years of age, and 61.4% had their first delivery in this same age group. Around 89.56% of Uyghur women in this study started having intercourse before being 20 years of age, which was twice as high as that of Chinese women in rural areas (40.9%) and seven times higher than women in urban China (12.5%).^13^ A study reported that Uyghur women's age at first marriage was delayed from 17.76 in the 1960s to 21.83 years old in the 1990s, reflecting the severity of risky sexual behavior in the early days of Xinjiang. We can then assume that in the 1960s, women started having sex at least 4 years earlier than women now. Cervical cancer risk is twice as high in women married before 16 years of age compared to those who marry after 20 years of age. Another study on minorities in rural Guizhou China showed that mean age of women's first marriage was 19.9 ± 1.6 years old, which is older than in Uyghur women (16.8 ± 2.3 years), and mean age of first delivery was 20.2 ± 1.7 years, which is older than in the women in this study (19.2 ± 3.0 years).^14^

As soon as women are sexually active, the risk of infection with HPV increases dramatically. It was reported that the median time from first reported sex to acquisition of any HPV was 5 months in the Tanzania study, where girls were engaged in sex at 15 or 16 years old.^15^ This is similar to our study where most women were married at 15–19 years old. However, in this study, we enrolled women older than 20 years, and the majority of them married at 15–19 years old. Therefore, the status of HPV acquisition after the first sex was not available. More than half of the participants of the study delivered their first child at the age of 15–19 years old. This, along with having their sexual debut at an early age, put Uyghur women at high risk of contracting HPV earlier in their life. Uyghur women in rural areas have lower awareness regarding healthy sexual practices and personal hygiene, such that most of them reported that they or their husbands do not shower before engaging in sex nor use sordid sanitary napkins, toilet paper, etc. Moreover, the divorce rate has been rising in recent years, which means that they might change their sexual partners. All of these factors put these women at a higher risk of HPV infection and related diseases in the decade following their marriage. This might explain why the incidence of first HPV infection peaked at age 30–34.

The HPV infection rate was lower in our study than that reported in mainland China (18.0%).^16^ The HPV detection rate continued to rise from age 25 until age 39, declined slightly at age 40–44, and sharply increased after age 45 in this study. The data from Zhao report indicated that HPV prevalence declined in women older than 24 years old before increasing in women aged 35–39 years and then remained stable. A study from Tunisia indicated that the prevalence of HPV infection was highest among women aged <30 years, followed by a nonsignificant decline in HPV prevalence in women aged 30–50 years, and a new increase after 50 years of age.^17^ In all available literature, there is one prevailing point that HPV infection was low at age 25–40, but in our results, HPV prevalence kept rising from the starting line, showing a decline only in the age group of 40–44 years. This implies that immunodeficiency may exist in Uyghur women, which leads to early infection and later clearance in their lives. The abnormal cytology detection rate was 3.9%, which is lower than the 5.49% reported by Cheng^18^ in mainland China, but higher than the 3.3% reported by Al Eyd in the UAE.^15^ Even though the survey was conducted in a rural area of Xinjiang, all cervical smear specimens were transported to the Affiliated Tumor Hospital of Xinjiang Medical University and diagnosed by an expert. Therefore, the difference in cytology positive rate compared to that in the literature is possibly caused by population variation.

The detection rate of CIN2+ was 1.53%, and this detection rate was adopted in the study since the cervical cancer registry is not available in Xinjiang. Our result is lower than the 2.8% reported in a multicenter study in China^19^ and the 1.6% reported in Mongolia,^20^ but is higher than the 0.5% reported in Thailand.^19^ The highest CIN2+ detection rate was observed in the 35–39 years old age group, which is 5 years earlier from Zhao's report on 40–44 years of age. A hospital‐based retrospective study conducted in Xinjiang Cancer Hospital reported that most cases of cervical cancer occurred in women aged 31–60 years, with a peak in the age group of 41–50 years.^21^ Data from the National Office for Cancer Prevention and Control indicated that two peaks of incidence rate appeared in women aged 40–44 years (18.7/100,000) and 80–84 years (13.9/100,000). Furthermore, the invasive cervical cancer detection rate in the current study was 250/100,000 (0.25%), which is much higher than the reported incidence rate in China and other countries.^22^

From the results of age‐specific prevalence of HPV infection, cytological abnormalities, and CIN2+ detection rate, we can speculate that cytological abnormalities (35–39) occur 5 years later than HPV infection (30–34) in Uyghur women. Our data showed that the peak age of both HPV and CIN2+ in Uyghur women was 5 years earlier than the result from a nationwide large multicenter survey conducted by Zhao, which was 35–39 years old for HPV infection and 40–44 years old for CIN2+. The younger age of HPV infection and CIN2+ detection may be the consequence of early marriage in the Uyghur minority. This information reflects the earlier acquisition of HPV and subsequent CIN2+ in Uyghur women than in other populations in China, highlighting the necessity of earlier cervical cancer screening for Uyghurs.

The careHPV test was designed specifically for use in low‐resource settings to screen women aged 30 years and older. The price for careHPV will be negotiated to be feasible for each eligible country or organization. Moreover, careHPV allows treatment during the same screening visit. Studies^23^, ^24^, ^25^, ^26^ on careHPV test also demonstrated better performance compared to conventional screening methods. In our previous study,^8^, ^9^ primary screening by careHPV with reflex cytology was confirmed to be the best option for rural areas, where there is a severe lack of health resources and cytologists. Studies have shown that the sensitivity of careHPV is uniformly high in all ages, while that of Pap smear is substantially better in older women.^27^ The careHPV test showed better sensitivity (91.7%–93.1%), PPV (25.6%–29.4%), and NPV (93.2%–94.8%) among all age groups in this study than the data from India (sensitivity of 37.7%–70.1%, PPV 12.5%, and NPV 54.5%–98%), but specificity (22.4%–23.8%) was lower than that reported (97.4%). A relatively low specificity means more careHPV positive with less pathological positivity, which may be related to limited colposcopy and biopsy skill, leading to missing exact pathological tissues from the cervix. The careHPV test showed consistency in all three age groups (≥25, ≥30, and ≥35 years), and is preferable to the Pap test for Xinjiang Uyghur women of all ages.

In conclusion, Uyghurs in Xinjiang suffer a huge burden of cervical cancer incidence and mortality. The cervical cancer screening program in rural areas sponsored by the Chinese government uses VIA/VILI or Pap smears, which greatly contributes to early detection and early diagnosis of disease in rural Xinjiang. Nevertheless, HPV DNA testing, for example, careHPV, showed more promising value in global screening projects. The study conducted by our research team also offered strong approval for the new screening protocol. Being an accurate, fast, feasible, and cheaper method, careHPV is the best choice for less developed regions in China. High risk factors, including early marriage and early delivery, are prevalent in the Uyghur community. HPV infection will occur soon after the first sexual intercourse, and HPV prevalence in Uyghur women in this study increased rapidly after baseline age. Furthermore, the HPV detection rate in our study increased starting from 25 years of age and showed a first peak at 30–34 years, while the first peak of CIN2+ was at 35–39 years. Moreover, the careHPV test showed similar performance among all age groups with different starting ages. In summary, the screening policy for Uyghur women may adopt the careHPV test as the primary screening method. Furthermore, the screening program may start at 30 years of age, which is 5 years earlier than the age of 35 years old recommended by the World Health Organization for low‐resource settings and by the Chinese government. In that case, more HPV‐infected Uyghur women with cervical lesions could be discovered without delay. Early detection and diagnosis, which are the core targets of cervical cancer screening, could be better achieved. To improve on this study, further health economic evaluations are needed to justify our findings.

## ETHICS STATEMENT

The study protocol has been approved by the Ethics Committees of The Affiliated Tumor Hospital of Xinjiang Medical University. Approval No: G‐201204.

## CONFLICT OF INTEREST

The authors declare no potential conflict of interest.

## Data Availability

All data models used during the study are available from the corresponding author upon reasonable request.

## References

[1] Bray F , Ferlay J , Soerjomataram I , et al. Global cancer statistics 2018: GLOBOCAN estimates of incidence and mortality worldwide for 36 cancers in 185 countries. CA Cancer J Clin. 2018;68(6):394‐424. 30207593 10.3322/caac.21492

[2] Women's health in rural China. Lancet. 2009;374:358. doi:10.1016/S0140-6736(09)61394-5 19647592

[3] The population census of the Peoples Republic of China. 2010. Accessed July 4, 2014. http://www.stats.gov.cn/tjsj/pcsj/rkpc/6rp/indexch.htm

[4] Qiao Y‐L , Zhao YQ . Cervical cancer epidemiology and prevention. Chin J Obstet Gynecol Pediatr. 2015;11(2):1–6. doi: 10.3877/cma.j.issn.1673-5250.2015.02.001

[5] Hou J . Study on marriage and family status of Xinjiang minority women. J Xinjiang Univ. 1996;24(3):20‐32.

[6] Saslow D , Solomon D , Lawson HW , et al. American Cancer Society, American Society for Colposcopy and Cervical Pathology, and American Society for Clinical Pathology screening guidelines for the prevention and early detection of cervical cancer. Am J Clin Pathol. 2012;137:516‐542. 22431528 10.1309/AJCPTGD94EVRSJCG

[7] Goldie SJ , Kuhn L , Denny L , et al. Policy analysis of cervical cancer screening strategies in low resource settings. JAMA. 2001;285(24):3107‐3115. doi: 10.1001/jama.285.24.3107 11427139

[8] Mijiti P , Abulizi G . Cross‐sectional study on uyghur cervical cancer in Hotan Karkax County, Xinjiang. J Xinjiang Med Univ. 2014;9:10 (doctoral dissertation).

[9] Tuerxun G , Yukesaier A , Lu L , et al. Evaluation of careHPV, cervista human papillomavirus, and hybrid capture 2 methods in diagnosing cervical intraepithelial neoplasia grade 2+ in Xinjiang Uyghur women. Oncologist. 2016;21(7):825‐831. 27317575 10.1634/theoncologist.2015-0447 PMC4943388

[10] Abulizi G , Chen JX , Mikeremu A . The spectrum of HPV infection in Xinjiang Uighur women with cervical cancer. Tumor. 2007;27(5):379‐382. doi: 10.3781/j.issn.1000-7431.2007.05.011

[11] Zhao N , Zhao FH , Gao XH , et al. Risk factors of high‐risk human papillomavirus infection in urban and rural area China. Chin J Cancer Prev Treat. 2011;18(16):1225‐1229.

[12] Sogukpınar N , Saydam BK , Can HO , et al. Assessment of cervical cancer risk in women between 15 and 49 years of age: case of Izmir. Asian Pacific J Cancer Prev. 2013;14(3):2119‐2125. 10.7314/apjcp.2013.14.3.2119 23679329

[13] Rui EX . Analysis on marriage and family planning of Uyghur and Chinese women in Hami Xinjiang. J Xinjiang Med Inst. 1994;17(1):36‐38.

[14] Wei YP , Zhao SP , Wei P , et al. Prevelance of high risk human papillomavirus infection in women in rural minority of Guizhou province. Matern Child Health Care China. 2010;25(32):4727‐4729.

[15] Zhao FH , Lewkowitz AK , Hu SY , et al. Prevalence of human papillomavirus and cervical intraepithelial neoplasia in China: a pooled analysis of 17 population‐based studies. Int J Cancer. 2012;131(12):2929‐2938. doi:10.1002/ijc.27571 22488743 PMC3435460

[16] Ardhaoui M , Ennaifer E , Letaief H , et al. Prevalence, genotype distribution and risk factors for cervical human papillomavirus infection in the Grand Tunis region, Tunisia. PLoS One. 2016;11(6):e0157432. doi:10.1371/journal.pone.0157432 27299955 PMC4907453

[17] Xue C , Jingmin Z , Jie J , et al. Analysis on cervical cancer screening of 65613 physical examination female in Nanjing. China Modern Doctor. 2014;52(33):72‐74.

[18] Al Eyd GJ , Shaik RB . Rate of opportunistic pap smear screening and patterns of epithelial cell abnormalities in pap smears in Ajman, United Arab Emirates. Sultan Qaboos Univ Med J. 2012;12:473‐478. 23275844 10.12816/0003173 PMC3523997

[19] Dondog B , Clifford GM , Vaccarella S , et al. Human papillomavirus infection in Ulaanbaatar, Mongolia: a population‐based study. Cancer Epidemiol Biomarkers Prev. 2008;17(7):1731‐1738. doi: 10.1158/1055-9965.EPI-07-2796 18628425

[20] Sukvirach S , Smith J , Tunsakul S , et al. Population‐based human papillomavirus prevalence in Lampang and Songkla, Thailand. J Infect Dis. 2003;187:1246‐1256. 12696004 10.1086/373901

[21] Zhu XY , Qiu J , Lai XJ . An analysis of in‐patients cancer type distribution in a period of 10 years in Xinjiang cancer hospital. Cancer. 2004;13(11):712‐714. doi: 10.3969/j.issn.1004-0242.2004.11.011

[22] Globecan. Estimated cancer incidence, mortality and prevalence worldwide. 2012. Accessed February 2, 2015. http://Globecan.iarc.fr/Pages/fact_sheet_cancer.aspx?cancer=cervix

[23] Qiao Y‐L , Sellors JW , Eder PS , et al. A new HPV‐DNA test for cervical‐cancer screening in developing regions: a cross‐sectional study of clinical accuracy in rural China. Lancet Oncol. 2008;9:929‐936. 18805733 10.1016/S1470-2045(08)70210-9

[24] Jeronimo J , Bansil P , Lim J , et al. A multicountry evaluation of careHPV testing, visual inspection with acetic acid, and papanicolaou testing for the detection of cervical cancer. Int J Gynecol Cancer. 2014;24:576‐585. 24557438 10.1097/IGC.0000000000000084 PMC4047307

[25] Labani S , Asthana S , Sodhani P , et al. CareHPV cervical cancer screening demonstration in a rural population of north India. Eur J Obstet Gynecol Reprod Biol. 2014;176:75‐79. 24685404 10.1016/j.ejogrb.2014.03.006

[26] Ngou J , Magooa MP , Gilham C , et al. Comparison of careHPV and hybrid capture 2 assays for detection of high‐risk human Papillomavirus DNA in cervical samples from HIV‐1‐infected African women. J Clin Microbiol. 2013;51:4240‐4242. 24108613 10.1128/JCM.02144-13 PMC3838057

[27] Arbyn M , Verdoodt F , Snijders PJF , et al. Accuracy of human papillomavirus testing on self‐collected versus clinician‐collected samples: a meta‐analysis. Lancet Oncol. 2014;15:172‐183. 24433684 10.1016/S1470-2045(13)70570-9

